# Development of a Clinical Practice Guideline for Lower Limb Amputees. A Knowledge Translation Process in a Middle Income Country

**DOI:** 10.3389/fresc.2022.873436

**Published:** 2022-05-03

**Authors:** Ana Maria Posada-Borrero, Daniel Felipe Patiño-Lugo, Jesus Alberto Plata-Contreras, Juan Carlos Velasquez-Correa, Luz Helena Lugo-Agudelo

**Affiliations:** Physical and Rehabilitation Department, School of Medicine, University of Antioquia, Medellín, Colombia

**Keywords:** implementation, clinical practical guidelines, lower limb amputation, knowledge translation (KT), rehabilitation

## Abstract

**Background and Aim:**

Knowledge translation processes are necessary for improving patients' and communities' health outcomes. The aim of this study was to systematically develop evidence-based recommendations for people over 16 years of age who are in risk for or have suffered a lower limb amputation for medical reasons (vascular, diabetes mellitus) or trauma (civilian or military trauma) in order to improve function, quality of life, decrease complications and morbidity.

**Methods:**

Following the Grading of Recommendations Assessment, Development and Evaluation (GRADE) approach we developed a Clinical Practice Guideline (CPG) for lower limb amputees with funding from the Ministry of Health in Colombia and participation of a multidisciplinary group. We included patients' preferences. Based on the scope, purposes and objectives the questions were elaborated with the PECOT strategy. The evidence search was performed for each question in the main databases: Cochrane Library, Embase and PubMed, without time limit or language restriction. Teams were formed with thematic experts and clinical epidemiologists to review the clinical studies, describe the evidence, and evaluate the quality of the body of evidence with the GRADE methodology. The recommendations were made according to the judgments proposed by the GRADE working group. We conducted a stakeholder's dialogue as a mechanism for the external validation of the guideline implementation.

**Results:**

The CPG included 43 recommendations related to the diagnosis, surgical treatment, rehabilitation, prescription and adaptation of the prosthesis. They were strong in favor 37.2, weak in favor 53.5, strong against 2.3, Weak against 7.0%. Quality of evidence was high in 0, moderate in 11.6, low in 58.1, and very low 30.2%.

**Discussion:**

In 93% of the recommendations, the quality of the evidence was between low and very low. This is why it was so important to validate and discuss each recommendation with an expanded multidisciplinary group. The research group identified 25 interventions and five milestones to be prioritized in the implementation and in the stakeholder's dialogue participants identified opportunities and barriers for implementation of recommendations.

**Conclusion:**

It is necessary to develop a national policy for implementation strategies of CPG recommendations that promotes the necessary arrangements for the provision of services for diagnosis, treatment, and rehabilitation of individuals with amputations.

## Background

Evidence-based clinical practice guidelines (CPG) are a fundamental tool for reforming medical care and strengthening health systems to achieve better health outcomes for patients and their communities (1, 2). However, despite the rigorous systematic synthesis of the scientific evidence contained in high-quality CPG, not all of them can be easily and directly translated into practice (3, 4).

In 2008, the Colombian Ministry of Health and Social Protection (MoH), financed the development of the methodological guideline for the development of evidence-based CPG in Colombia, this guideline was updated in the year 2014 (5). Between 2008 and 2016, the MoH in Colombia financed and convened the elaboration of 58 national CPG that were elaborated with the best methodological standards, by professionals from recognized universities in Colombia, with the participation of scientific associations, healthcare professionals and patients and caregivers. The purpose was to reduce unjustified variability in medical practice, improve the efficient management of resources, and be able to offer patients the most effective and safest interventions (6). A CPG implementation manual was also developed, with general suggestions about how to implement these CPGs in the different healthcare provider institutions (7).

In 2013, through a call of the Administrative Department of Science, Technology and Innovation (nowadays Minciencias) and financed by the MoH, we developed the “Clinical Practice Guideline for diagnosis and preoperative, intraoperative and postoperative treatment of the amputee, the prescription of the prosthesis and comprehensive rehabilitation” (8). An interdisciplinary group involved in the care of amputee patients from different cities in Colombia participated in its preparation. This guideline was updated in 2018.

One of the most consistent findings of clinical and health services research is the challenge to translate research evidence into practice (1). This have been reported around the world in different income level countries and in different sectors of care, such as primary or specialty care (1).

The US National Center for Dissemination Research on Disability defines knowledge transfer as “The collaborative and systematic review, evaluation, identification, aggregation, and practical application of high-quality research on disability and rehabilitation by key stakeholders, in order to improve the lives of people with disabilities” (9). This definition recognizes that there is a wide range of stakeholders for knowledge transfer, including policy makers; health providers; end users, researchers and industry. It is important that these transfer processes are implemented, especially in low and middle-income countries, strengthening the rehabilitation of people with disabilities.

The aim of this study was to systematically develop evidence-based recommendations for people over 16 years of age who are in risk for or have suffered a lower limb amputation for medical reasons (vascular, diabetes mellitus) or trauma (civilian or military trauma) in order to improve function, quality of life, decrease complications and morbidity.

## Materials and Methods

### Participants

The main guideline developer group consisted of 14 people, including physicians, physiatrists, orthopedists, vascular surgeons, experts in prosthetics, psychiatrists, physiotherapists, occupational therapists, clinical epidemiologists, public health doctors, economists, a documentary librarian, and undergraduate and postgraduate students. A group of professionals from different universities and scientific societies validated the different stages of the process. A focus group of 24 people with amputations of different causes and their relatives were linked to the process in two moments, when the questions were chosen and at the end of the recommendations. The developer group received a training process with different international centers as the McMaster University, the *National Institute for Health and Clinical Excellence* (NICE) and the *New Zealand Guidelines Group*. The users of the CPG are all the professionals who were involved in the development: surgeons, physiatrists, other professionals in the area of rehabilitation, insurers, health providers and political decision makers.

### Ethical Aspects in the Development of the CPG

All the professionals who participated in the development made a declaration of interests at the beginning and each year. These are published as supplementary files within the CPG document (8).

The financing entity was the Ministry of Health and Social Protection, none of the people from this entity participated in the group developing the CPG. The Ministry carried out permanent monitoring to guarantee compliance with the methodology and schedules.

### GPC Search and Quality Appraisal

CPG for lower limb amputees were searched for, and an evaluation of quality was made with the AGREE II Instrument. Six CPG were evaluated independently by two professionals from the group. Only three with a score greater than 60 in the methodological domain were selected, which were used as information during development. (8).

### Prioritization of Outcomes and Elaboration of Questions

The development of the CPG followed the GRADE (Grading of Recommendations Assessment, Development and Evaluation) methodology (10, 11). For the elaboration of the recommendations of the CPG, within the guideline development group (GDG), a process of prioritization of the topics of interest was carried out and the most important were selected. Subsequently, clinical questions were formulated and a systematic review of the available evidence was made on each one. This process was done between 2014 and 2015. The main recommendations were updated in 2018.

Based on the scope, purposes and objectives of the guideline, the questions were elaborated with the PECOT strategy (Population, Exposure or intervention, Comparison, Outcomes and Time). Then the developer group and the patients independently rated the importance of each outcome from 1 to 9, according to the GRADE classification (11). According to the average scores of the developer group, the outcomes were classified as: critical (7-9), important non-critical (4-6) or not important (1-3). The evaluation of the quality of the body of evidence is done by selecting the critical and important outcomes.

### Literature Search Strategy

For each question, a list of MeSH terms was prepared according to the population, the intervention and the comparison. The evidence search was performed in the main databases: Cochrane Library, Embase and PubMed, and in secondary databases such as Lilacs/Bireme, Current Controlled Trials, TripDatabase and Google Scholar. There was no language restriction. For the selection of the evidence, inclusion criteria were established with respect to the methodological design, the population and the minimum quality characteristics. Systematic reviews and meta-analyses (secondary or aggregative studies), which analyzed primary studies related to the question, were initially sought. Additionally, clinical trials and observational studies were identified.

### Appraisal of the Quality of Evidence

The quality of the evidence was evaluated for systematic reviews and meta-analyses with the AMSTAR (12); for diagnostic studies the QUADAS (13); and with the STROBE for observational studies (14). The quality of the body of evidence was assessed according to the concepts of the GRADE methodology (11), by qualifying each outcome. This process was done by orthopedic doctors, physiatrists, and clinical epidemiologists, who were experts in the GRADE methodology. GRADE publications can be accessed on the website https://www.gradeworkinggroup.org/.

Systematic reviews of clinical trials start with high quality (level 1), while reviews of observational studies start with low quality (level 4). The aspects that can lower the quality of a randomized controlled trial are: Risk of bias, inconsistency of the results, indirect evidence, imprecision of the results and publication bias. Observational studies, although they can lower quality with the aforementioned aspects, also they can increase it if they include some favorable methodological aspects. The three aspects that can increase the quality of are the presence of a large effect size (Relative Risk, >2.0 or <0.5); evidence of a gradient dose-response relationship and the absence of residual bias or confounding factors (15–20).

The quality of the evidence is related to the confidence that the true effect is close to the estimated effect. Four levels are defined: very low, low, moderate and high (11). Most of the quality of the evidence for this guideline was low or very low quality of evidence.

### From Evidence to Recommendation

Following the GRADE system, the elaboration of the recommendations does not only take into account the quality of the evidence, but also a series of aspects or judgments based on the following criteria: The priority of the problem, the magnitude of the desirable and undesirable effects, the certainty of the evidence, the values of the interested parties, the balance between desirable and undesirable effects, the resources required, the cost-effectiveness, equity, acceptability and feasibility. With these criteria, a summary table of judgments was created and the direction and strength of the recommendation were defined (21). The strength of the recommendations is rated in four categories: Strong (recommended to do), weak in favor (suggested to do), strong against (recommended not to do), weak against (suggested not to do) (21). During face-to-face sessions with the entire guideline development group, the evidence for each question, the quality of the body of evidence, and the judgments were presented. With the foregoing, a recommendation was drawn up that was subsequently validated by an extended group with thematic experts and representatives of scientific societies and universities.

### Economic Evaluations

Five economic evaluations were made during the development of the CPG to assess the cost-effectiveness of five of the interventions and help the guideline development group in the decision-making.

### Consumer Preferences

In the development of a CPG, it is recommended including the perspective of the patients for the preparation of the recommendations. Thus, people with lower limb amputation were invited to define their priorities in three categories: complications, activities and prosthetic adaptation; using the GRADE methodology. In addition, their preferences of the treatment options in the recommendations with greater uncertainty and with low quality of evidence were evaluated.

Between July and November 2014, people with amputation in two institutions that provide health services in two cities of the country were invited. The inclusion criteria were people from 18 to 65 years old, who had a major lower limb amputation of any level and cause and who could attend a meeting with the researchers. Children and upper limb amputees were excluded. A convenience sampling was used, with the people who responded to the call. The objectives of the CPG and their participation, the instruments that were applied and doubts were resolved were explained at the meeting. In the group of 20 patients studied in one of the cities, the preferences of the CPG questions in which there was greater uncertainty at the time of presentation of the evidence synthesis were also evaluated.

## Results

### Literature Search

One search strategy is presented as an example in Table 1, for one of the surgical recommendations, elaborated during the updating of the CPG. All the other search strategies can be consulted in the complete document of the CPG (8).

**Table 1:** Example of the search strategy and results in data bases for one of the CPG questions in the 2018 update.

**DB**	**Search strategy**
PubMed 328	(Amputation[MeSH] OR Amputation, Traumatic[MeSH] OR traumatic amputat*[tiab]) AND (Lower Extremity[MeSH] OR Leg Injuries[MeSH] OR lower limb[tiab] OR LLA[tiab]) AND (Disarticulation[MeSH] OR Replantation[MeSH] OR Limb Salvage[MeSH] OR salvage[tiab] OR reconstruction[tiab] OR disarticulation[tiab]) AND ((“2015/01/01”[PDat]: “3000/12/31”[PDat]))
Embase 67	(‘amputation'/exp OR ‘amputation' OR ‘traumatic amputation'/exp OR ‘traumatic amputation' OR ‘diabetic foot'/exp OR ‘diabetic foot') AND (‘reimplantation'/exp OR ‘limb salvage'/exp) AND ([cochrane review]/lim OR [systematic review]/lim OR [controlled clinical trial]/lim OR [randomized controlled trial]/lim OR [meta-analysis]/lim) AND [2015-2018]/py
Cochrane 13	[Amputation] explode all trees OR [Amputation Stumps] explode all trees OR [Amputation, Traumatic] explode all trees OR [Amputees] explode all trees AND [Limb Salvage] explode all trees OR [Replantation] explode all trees. Since 2015

*PECOT question: In patients over 16 years old with severe lower limb trauma, is reconstruction of the limb compared to amputation at any level more effective and safer to achieve better function, return to work, reduce the need for additional surgical procedures, infection or residual pain in the first 12 months after surgery?*

### GPC Recommendations

**Figure 1:**
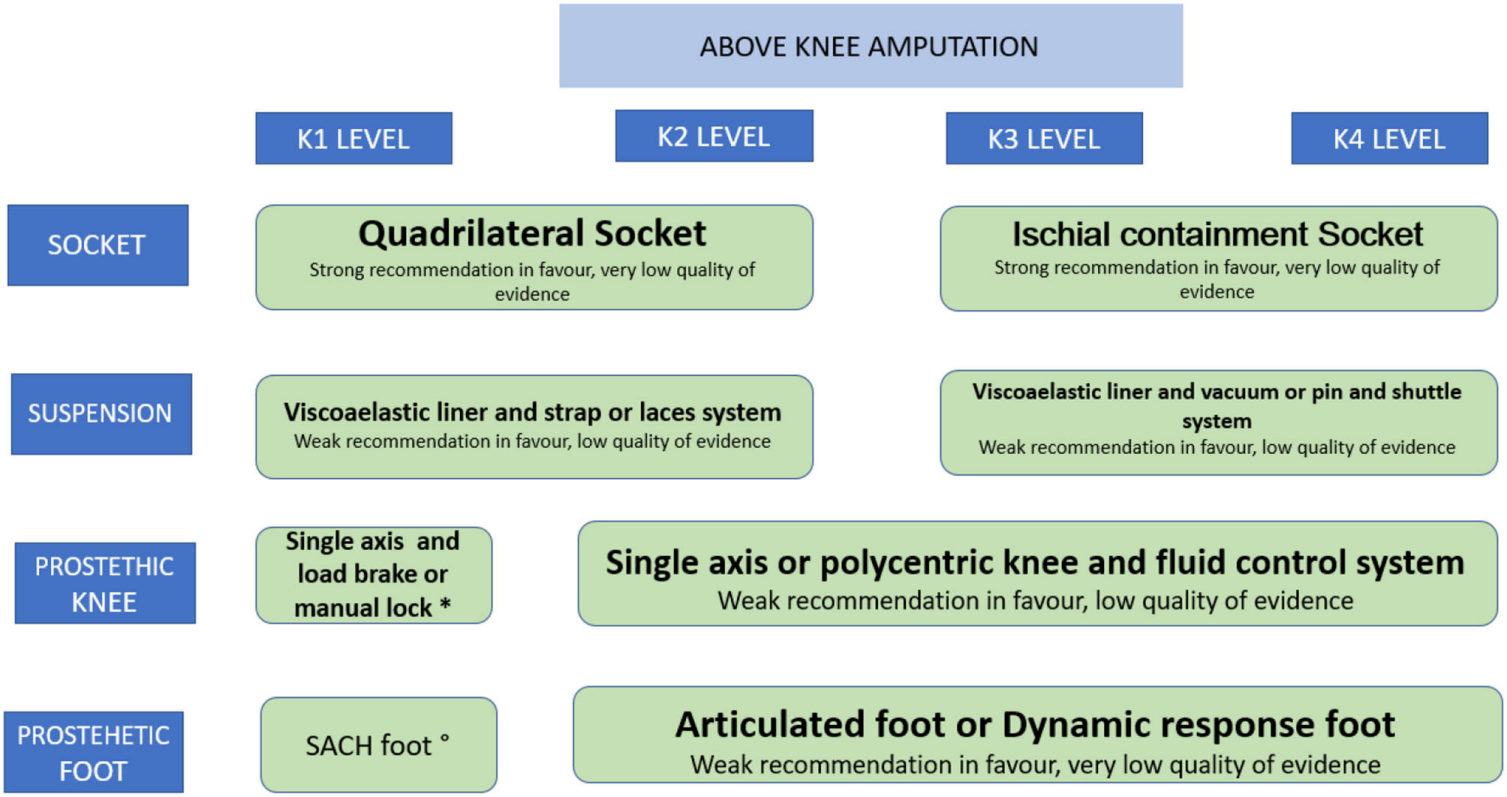
Recommendations for the prescription of the prosthesis in amputations above the knee. *Weak recommendation in favour. low quality of evidence. °Strong recommendation in favour. low quality of evidence.

**Figure 2:**
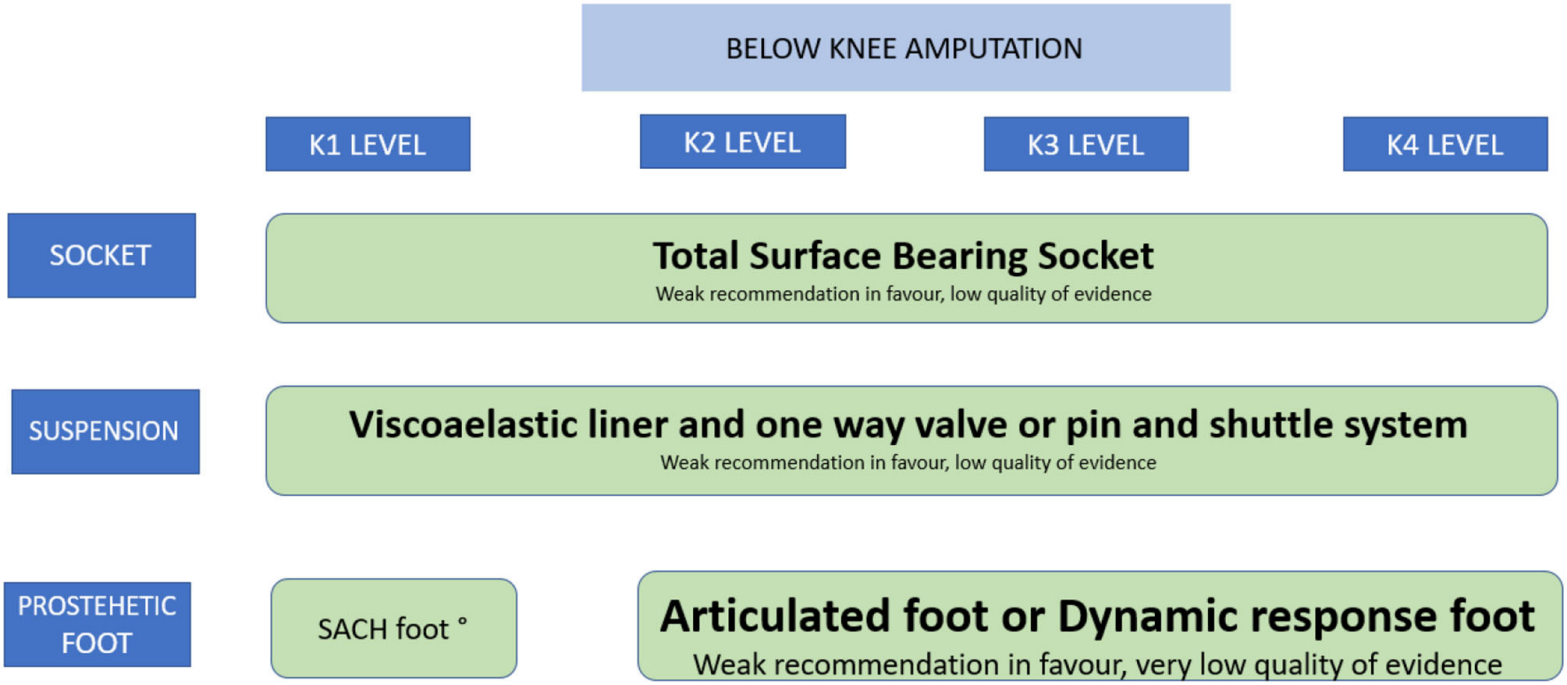
Recommendations for the prescription of the prosthesis in amputations below the knee. °Strong recommendation in favour. low quality of evidence.

Forty-three recommendations were made. Nine on the decision of amputation; five on preoperative interventions including: preoperative regional analgesia, cardiovascular reconditioning, psychological support, prophylactic antibiotics, and intraoperative tourniquet use; ten on amputation techniques; ten on the components of the prosthesis, feet, knees, sockets, liners, as well as orthoses for partial amputation and the adaptation of immediate postoperative prostheses. Nine for the post-prosthetic phase, including functioning scales to evaluate the use of prostheses; treatment for neuropathic and phantom limb pain; cardiopulmonary, physical and occupational rehabilitation; ergonomic adaptations and psychosocial interventions. Comprehensive rehabilitation compared to the usual care model was also evaluated and this was a strong recommendation in favor. The synthesis of the quality of the evidence and the strength of the recommendations can be seen in Table 2, and Figures 1, 2.

**Table 2:** Recommendations with quality of evidence and strength of recommendation (*n* = 43).

					**Recommendation**		**Quality of the evidence**		
		**Strong in favor**	**Weak in favor**	**Strong against**	**Weak against**	**Very low**	**Low**	**Moderate**	**High**
**AMPUTATION DECISION AND ITS LEVEL. TRAUMA**									
1	The use of any scale (MESS, NISSA, PSI, LSI and HFS-97) is not suggested in patients over 16 years old with lower limb trauma to define the type of intervention								
2	The use of any scale (MESS, NISSA, PSI, LSI and HFS-97) is not recommended in patients over 16 years old with lower limb trauma to predict function								
3	Soft tissue reconstructive procedures, flaps or grafts, are suggested for the treatment of soft tissue coverage defects of the amputation stump below the knee to preserve this joint and maintain a level of transtibial amputation								
4	Limb reconstruction is suggested in patients over 16 years old with severe lower limb trauma rather than amputation								
**AMPUTATION DECISION AND ITS LEVEL. VASCULAR**									
5	It is suggested to measure the transcutaneous oxygen tension to complement the surgeon's clinical decision.								
6	Plethysmography along with digital systolic blood pressure and ankle systolic blood pressure is suggested if transcutaneous oxygen tension is not available to supplement a surgeon's clinical assessment								
7	Two-stage amputation rather than single-stage amputation with primary closure is recommended for patients who require lower limb amputation secondary to moist necrotizing gangrene and severe infections								
**AMPUTATION DECISION AND ITS LEVEL. DIABETES**									
8	It is suggested to use the Texas or Wagner classification in patients with diabetic foot ulcers to predict the risk of amputation in clinical practice								
9	Transtibial amputation is suggested in patients over 16 years old who require amputation of the lower limb secondary to neuropathic or vascular disorders to reduce the risk of reamputation in the first 12 months								
**PREOPERATIVE INTERVENTIONS**									
10	Perioperative epidural analgesia is suggested in patients over 16 years old for lower limb amputation surgery to reduce acute stump and phantom limb pain in the postoperative period								
11	A preoperative cardiovascular reconditioning program is recommended in patients with vascular disease who are at risk of lower limb amputation.								
12	Preoperative psychological support is suggested in patients with vascular disease who are at risk of amputation.								
13	The use of prophylactic antibiotics is recommended for not longer than 24 h after amputation to prevent infection of the stump								
**AMPUTATION TECHNIQUES**									
14	The use of an intraoperative tourniquet is suggested in patients who require a transtibial amputation due to traumatic, ischemic or diabetic causes								
15	Amputation of the midfoot or hindfoot is suggested in patients with two or more rays affected due to ischemic causes or diabetes								
16	Performing a Syme amputation that allows adequate coverage, mobility, and function is suggested in patients who require a distal amputation due to vascular or metabolic etiology								
17	It is suggested that the choice of transtibial amputation flap be a matter of surgeon preference taking into account factors such as prior experience with a particular technique, the extent of non-viable tissue, and the location of pre-existing surgical scars								
18	The conventional technique (without distal tibiofibular bone bridge) is recommended instead of the modified Ertl (with tibiofibular bone bridge), in patients who require a transtibial amputation, due to traumatic, ischemic or diabetic causes								
19	It is recommended to guarantee adequate soft tissue coverage in the transtibial amputation stump in patients requiring amputation due to traumatic or vascular etiology, to allow an adequate balance of muscular forces, avoid shearing of the flaps and improve the stability of the stump within of the prosthesis; this coverage can be obtained with myodesis or myoplasty techniques								
20	A transfemoral amputation rather than a knee disarticulation is suggested for patients older than 16 years who require a lower-limb amputation and are not candidates for below-the-knee amputation								
21	Myodesis of the amputation stump is recommended in patients who require a transfemoral amputation due to traumatic or vascular etiology								
22	It is recommended when performing a transfemoral amputation to obtain a bony stump of at least 57% of the length of the contralateral femur in patients who require a transfemoral amputation for traumatic, ischemic or diabetic causes								
23	It is suggested to close the skin of the amputation stump in the lower limb with non-absorbable monofilament sutures, in patients who require amputation due to traumatic or vascular causes, to reduce the risk of surgical complications								
24	The use of closed suction drainage systems after definitive closure is not routinely suggested in patients who require amputation of the lower limb for traumatic, ischemic or diabetic causes, to reduce the risk of infection and the need for additional surgeries. by bruises or seromas								
**PROSTHETICS**									
25	The use of an immediate postoperative prosthesis is suggested in patients with lower limb amputation due to traumatic and vascular causes, to improve the remodeling of the stump								
26	Fitting an orthopedic insole or orthosis is recommended for people with partial foot amputations								
27	It is recommended for people with an amputation above or below the knee and a low expected functional level (K1/K2), the adaptation of a SACH foot								
28	The adaptation of an articulated foot or a dynamic response foot is suggested in people with higher activity requirements (K3/K4) or who must use the prosthesis on irregular or inclined surfaces, recommended by a specialist doctor with training in the area of prosthetics and social or environmental conditions make it possible								
29	The fitting of a full contact socket prosthesis with a silicone sleeve is suggested for below-knee amputees								
30	A prosthesis with a full contact socket with a liner in silicone, copolymer or polyurethane is suggested for people with amputation below the knee. The use of a vacuum valve or a pin and lock system must be individualized								
31	In people with amputation above the knee and an expected functional K1 level, the adaptation of a monocentric knee with manual locking or with a load brake is suggested, in K2, K3, and K4 a monocentric or polycentric fluid control								
32	In people with knee disarticulation and an expected functional level of K1, the adaptation of a mechanical polycentric knee for knee disarticulation is suggested; and in K2, K3 and K4 a fluid control polycentric								
**REHABILITATION**									
33	In people with an above-knee amputation and moderate or high functional levels, the adaptation of one of the ischial containment socket variants is recommended. In people with low functional levels, the adaptation of a quadrilateral socket is recommended								
34	For above-knee amputees, individualized adaptation of a suspension system is recommended based on the patient's functional capabilities and residual limb condition								
35	In patients with lower limb amputations due to trauma, vascular or diabetes, the use of one or more of the scales (PEQ-MS, 2MWT, TUG and SIGAM) is suggested for the evaluation of musculoskeletal function and movement								
36	The use of the Houghton Scale is suggested to assess prosthetic adaptation in patients who had a lower limb amputated due to traumatic, vascular or diabetic causes								
37	It is not suggested to use neuropsychological therapies (mirror therapy) in patients with lower limb amputation due to traumatic, vascular or diabetic causes, for the improvement of phantom limb pain								
38	Pregabalin is recommended first, followed by gabapentin, amitriptyline, and duloxetine as monotherapy, in amputated patients due to trauma, vascular causes, or diabetes to improve neuropathic pain								
39	The implementation of a cardiopulmonary rehabilitation program is suggested in patients with lower limb amputation due to traumatic, vascular or diabetic causes								
40	The implementation of a physical rehabilitation program that includes muscle strength, joint mobility, balance, gait, physical reconditioning is recommended in patients with lower limb amputation, due to traumatic, vascular or diabetic causes								
41	Occupational rehabilitation and ergonomic adaptations are recommended in patients with lower limb amputation due to trauma, vascular or diabetes, to improve functioning and facilitate return to work or an occupation								
42	Post-prosthetic psychosocial interventions in which the patient and their family are involved are recommended in patients who have had a lower limb amputated due to traumatic, vascular or diabetic causes								
43	The implementation of a comprehensive rehabilitation process is recommended: cardiopulmonary, musculoskeletal, psychosocial, activities of daily living and for work, in patients with lower limb amputation, due to traumatic, vascular or diabetic causes								

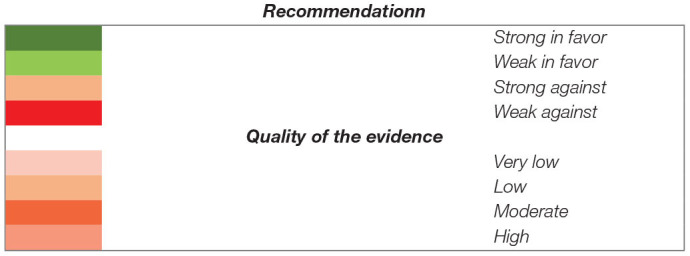

The distribution of the 43 recommendations, according to the quality of the evidence, was: high 0%, moderate 7.5%, low 57.5%, very low 35%. And according to the strength of the recommendations, their distribution was: Strongly in favor 37.2%; weak in favor 53.48%; strong against 2.32%; weak against 6.97%. In updating the prioritized questions, a recommendation changed from Weak in favor to Strong in favor, leaving the distribution as follows: Strong in favor 58.3%, weak in favor 41.6%.

### Consumer Preferences

As part of the participation of patients in the development of the CPG, they were invited to assess the importance of each outcome of the recommendations (22). Patients chose stump infection in 31.7%, death in 22%, stump reoperation in 22%, and phantom pain in 12.2% as the most important outcomes for them. The most important activities in the rehabilitation process were walking in 51.2%, returning to work in 17.1%, having a good quality of life in 14.6% and participating in social activities in 7.3%. Twenty patients participated in the evaluation of preferences. Of them, 95% prefer to keep the knee to a transfemoral amputation, 60% prefer amputation in the first surgery than reconstruction, 75% agree with the need for psychological support, 85% agree with a supervised exercise plan after amputation and, only 45% agree with the use of an immediate prosthesis (22).

### Economic Evaluations

The results of the first economic evaluation concluded that, after a careful selection of patients and intervention by a multidisciplinary team, limb reconstruction was a dominant strategy compared to primary amputation in the long term (23).

In the second evaluation, the adaptation of an articulated foot was not a cost-effective strategy compared to a SACH foot, in patients with a low level of activity (8).

In the third, in a sample of 113 patients analyzed in a cross-sectional study, the total contact socket was a cost-effective strategy compared to a patellar-tendon-bearing (PTB) type (8). However, it is not possible to determine if this result can be extrapolated to other populations of patients with amputations below the knee in Colombia.

In the fourth economic evaluation, Pregabalin was found to be the strategy with the greatest net benefit, so it can be considered first-line treatment of phantom pain or residual pain in lower limb patients with amputation (24). Gabapentin and amitriptyline had similar, albeit lower, net benefits and could also be considered at the discretion and experience of the treating physician. More research is needed on the effectiveness of medications in patients with lower limb amputation.

In the fifth economic evaluation on the cost-effectiveness of prophylactic antibiotics, it was found that this is a dominant strategy and it is unlikely that the uncertainty surrounding the costs and benefits changes the results, the use of this intervention is recommended in Colombia (25).

### Implementation Plan

As a final result of the CPG, the implementation process was described based on planning, implementation activities, monitoring and evaluation. Structure, process and outcome indicators were defined. Structure indicators were the availability of surgical and rehabilitation services. Process indicators were the proportion of patients with prostheses and in a rehabilitation program, according to the recommendations of the guidelines. Outcome indicators were the proportion of patients with reamputation, the proportion of patients adapted to prosthesis, and the proportion of professionals who follow the recommendations of the CPG.

## Discussion

This article described the methods and results of the elaboration of an evidence-based CPG for the care of people with lower limb amputations.

The elaboration of the CPG started with the formation of a multidisciplinary group that received training in methods form international and national universities and centers. The guideline included 43 recommendations, where nine were about the amputation decision and the level of amputation; five on preoperative interventions; 10 on amputation techniques; 10 on prosthetic components and nine on post-prosthetic rehabilitation. In 93% of the recommendations, the quality of the evidence was between low and very low. This is why it was so important, on a permanent basis, to validate and discuss each recommendation with an expanded multidisciplinary group with experience in treating lower-limb amputees. The socialization was carried out with different actors interested in the care of these patients.

This CPG was evaluated by international experts using the AGREE II instrument, with a score of 94/100, and was recommended for its implementation in Colombia. During the development, other CPG were evaluated, in which the scope and purpose domain had scores between 65.3 and 98.6%; in the stakeholder involvement between 54.1 and 97.2%; in the rigor of development between 25.0 and 85.9%; in the domain of clarity of presentation between 62.5 and 95.8; in applicability between 18.8 and 93.8%; and in editorial independence between 14.1 and 7.9% (8). This is in agreement with an article that evaluated the quality of the evidence of four CPG with 217 recommendations and found that the quality of the evidence was low (26). In addition, in the rehabilitation questions only 6.9% came from randomized clinical trials (RCT), systematic reviews or meta-analyses.

Although there were three CPG that had a score >60 in the methodological domain of the AGREII rating (27–29), they were not adapted because many of the questions raised by the developer group did not coincide with the questions of the guidelines. And the second reason was because the methodological guideline of Colombia (7) recommends that the guidelines in Colombia must be developed with the GRADE methodology and the guidelines did not follow this methodology at the time of the CPG search.

The research group identified 25 interventions and five milestones to be prioritized in the implementation. The milestones included re-amputation, reinterventions due to infectious processes, prosthetic adaptation, return to work and independence in activities of daily living (30).

We conducted a stakeholder's dialogue as a mechanism for the external validation of the Guideline implementation (31). Fifty-four actors participated in this forum, including: professionals from the MoH, representatives from health insurance companies, health provider institutions, academic professionals, scientific associations and thematic experts from different areas, patients, undergraduate and postgraduate students. In this dialogue participants emphasized the need to build integrated rehabilitation programs that are close to the patients in order to guarantee access to the health services with the minimum displacement of the patient. It is important to include care in the area of mental health. Successful prosthetic adaptation also depends on family support, training in activities of daily living, modification of the home and community environment, and occupational reintegration. Users must be guaranteed that they have sufficient and timely information, and continuous training, so that they are active actors in their surgical and rehabilitation process, through knowledge of their rights.

Insurers must recognize their responsibility in the care, rehabilitation and risk management of their insured population. Extramural actions must be included that allow the decentralization of rehabilitation services.

For the stakeholder participants it is important to have an information system for all personnel in charge, and to be able to measure the quality of care and the outcomes in patients. The referral and counter-referral process should be strengthened so that patients residing in rural and dispersed areas can access services in the main cities. In addition, implement a system in which patients are referred to centers where their needs can be effectively responded to. It is important for the country to involve these aspects in medical training and related professions, as well as continuing education for professionals involved in patient care, including evidence-based medicine and CPG training.

Several facilitators must be involved to improve patient accessibility such as technological tools, telemedicine and tele-rehabilitation (32, 33). These strategies were strengthened during the SARS2 COVID 19 pandemic.

The most important barriers and facilitators found in a qualitative study made by the research group and that were decisive for the implementation of the CPG for amputees included challenges related to the governance and financial arrangements of the Colombian health systems (34). For example, the Colombian health system could mandate that health care institutions establish procedures to adapt CPGs for amputee patients. At the time, health institutions are only required to have CPG for the most 10 prevalent health conditions; and amputations do not meet that requirement. Regarding financial arrangements, policymakers could ensure that access to the promotion, prevention, diagnosis, treatment, and rehabilitation of individuals with amputations does not depend on the type of patient insurance (34). In the systematic meta-review, there was greater emphasis on the barriers related to professionals, such as lack of credibility in the evidence, lack of training in CPG, the absence of a leader, and difficulties with the work team. Patients identified the lack of information from health professionals as difficulties, expressing the need for prostheses to be adapted according to their context (35).

The results of investments in research and training of health personnel to improve the quality of care are not being taken into account in health practice settings and many patients are not receiving the best possible care. This represents a gap between medical advances and clinical practice. Similar findings have been reported around the world in both developed and developing settings, in primary and specialty care (1).

### Implications for Practice and Policy

For health professionals in charge of caring for amputees, it is important to emphasize the need for patients to receive the most effective and safe interventions. Patients need to receive this intervention in time to reduce functional limitations and achieve occupational reintegration and social participation for amputees.

Rehabilitation services must be comprehensive and available in a place that is close to patients to reduce the possibility of loss in the continuity of care. In the country, travel is paid for by patients and their families and this can be an even greater barrier if they must go to different places for their treatment. Comprehensive rehabilitation must involve mental health aspects to prepare amputees in the phase of acceptance and mourning for the loss of their limb and provide support in rehabilitation. Also, comprehensive rehabilitation must include physiatrist care, physical and occupational therapy, cardiopulmonary rehabilitation, psychology care and very importantly access to the prosthesis and its adaptation. All of the above will make possible for amputees to return to their usual occupation and integrate into society.

Professionals must have the necessary training, time and incentives to achieve a change in professional practice.

### Implications for the Health System and Policies

In Colombia, administrative procedures with insurers companies are lengthy and amputee patients must take multiple steps to obtain approval for each of the interventions and devices necessary for their rehabilitation (36, 37). The rehabilitation program should be approved as a package of interventions based on the recommendations of the CPG.

### Limitations and Strengths

One limitation of the study is not having the final results of the implementation project to make better analyzes of the situations presented.

## Conclusions

It is not enough to prepare a CPG of very good quality, to elaborate a comprehensive health care pathway and to assess the barriers and facilitators for recommendations implementation, to improve the healthcare process of people with lower limb amputations in Colombia. It is necessary to develop a national policy that promotes the necessary arrangements for the provision of services as coordination of care amongst different providers, communications between them, continuity of care, package of care, referral systems, shared care, multidisciplinary teams, planning the transition of care from hospital to the community, health information systems development. Financial and governance arrangements and finally implementation strategies targeted at healthcare organizations, at healthcare workers, and in a specific type of practice.

## Author Contributions

All authors certify that they have participated sufficiently in the work to take public responsibility for the content, including participation in the concept, design, analysis, writing, or revision of the manuscript.

## Funding

Article financed by the Ministry of Science, Technology and Innovation - Minciencias, under contract 738 of 2017, project called: Efectividad de una estrategia basada en Telesalud para mejorar la implementación de la guía de práctica clínica para el diagnóstico y tratamiento preoperatorio, intraoperatorio y postoperatorio de la persona amputada, la prescripción de la prótesis y la rehabilitación integral en instituciones de salud en Antioquia: un estudio de intervención aleatorizado por conglomerados de hospitales.

## Conflict of Interest

JP-C is the technical director at Mahavir K-mina, a non-governmental organization that manufactures lower limb exoskeletal prostheses. The remaining authors declare that the research was conducted in the absence of any commercial or financial relationships that could be construed as a potential conflict of interest.

## Publisher's Note

All claims expressed in this article are solely those of the authors and do not necessarily represent those of their affiliated organizations, or those of the publisher, the editors and the reviewers. Any product that may be evaluated in this article, or claim that may be made by its manufacturer, is not guaranteed or endorsed by the publisher.
